# Temporal gene expression profiling during early-stage traumatic temporomandibular joint bony ankylosis in a sheep model

**DOI:** 10.1186/s12903-024-03971-x

**Published:** 2024-02-28

**Authors:** Tong-Mei Zhang, Kun Yang, Mai-Ning Jiao, Yan Zhao, Zhao-Yuan Xu, Guan-Meng Zhang, Hua-Lun Wang, Su-Xia Liang, Ying-Bin Yan

**Affiliations:** 1 Tianjin Medical University Cancer Institute & Hospital, National Clinical Research Center for Cancer, West Huan-Hu Road, Ti Yuan Bei, Hexi District, Tianjin, 30060 PR China; 2 Tianjin’s Clinical Research Center for Cancer, West Huan-Hu Road, Ti Yuan Bei, Hexi District, Tianjin, 30060 PR China; 3 Key Laboratory of Cancer Prevention and Therapy, Tianjin, West Huan-Hu Road, Ti Yuan Bei, Hexi District, Tianjin, 30060 PR China; 4 Tianjin Medical University, 22 Qi-xiang-tai Road, Heping District, Tianjin, 300070 PR China; 5 Department of Oromaxillofacial-Head and Neck Surgery, China Three Gorges University Affiliated Renhe Hospital, 410 Yiling Ave, Hubei, 443001 PR China; 6 Department of Oral and Maxillofacial Surgery, Weifang people’s Hospital, 151 GuangWen Street, KuiWen District, Weifang, ShanDong Province 261000 PR China; 7 Department of Oromaxillofacial-Head and Neck Surgery, Tianjin Stomatological Hospital, School of Medicine, Nankai University, 75 Dagu Road, Heping District, Tianjin, 300041 PR China; 8 Tianjin Key Laboratory of Oral and Maxillofacial Function Reconstruction, 75 Dagu Road, Heping District, Tianjin, 300041 PR China; 9 Department of Oral and Maxillofacial Surgery, Jining Stomatological Hospital, 22 Communist Youth League Road, Rencheng District, Jining, ShanDong Province 272000 PR China; 10 Department of Operative Dentistry and Endodontics, Tianjin Stomatological Hospital, School of Medicine, Nankai University, 75 Dagu Road, Heping District, Tianjin, 300041 PR China

**Keywords:** Temporomandibular joint, Mandibular condyle, Ankylosis, Microarray analysis, Trauma, Sheep, Animal

## Abstract

**Background:**

Investigating the molecular biology underpinning the early-stage of traumatic temporomandibular joint (TMJ) ankylosis is crucial for discovering new ways to prevent the disease. This study aimed to explore the dynamic changes of transcriptome from the intra-articular hematoma or the newly generated ankylosed callus during the onset and early progression of TMJ ankylosis.

**Methods:**

Based on a well-established sheep model of TMJ bony ankylosis, the genome-wide microarray data were obtained from samples at postoperative Days 1, 4, 7, 9, 11, 14 and 28, with intra-articular hematoma at Day 1 serving as controls. Fold changes in gene expression values were measured, and genes were identified via clustering based on time series analysis and further categorised into three major temporal classes: increased, variable and decreased expression groups. The genes in these three temporal groups were further analysed to reveal pathways and establish their biological significance.

**Results:**

Osteoblastic and angiogenetic genes were found to be significantly expressed in the increased expression group. Genes linked to inflammation and osteoclasts were found in the decreased expression group. The various biological processes and pathways related to each temporal expression group were identified, and the increased expression group comprised genes exclusively involved in the following pathways: Hippo signaling pathway, Wnt signaling pathway and Rap 1 signaling pathway. The decreased expression group comprised genes exclusively involved in immune-related pathways and osteoclast differentiation. The variable expression group consisted of genes associated with DNA replication, DNA repair and DNA recombination. Significant biological pathways and transcription factors expressed at each time point postoperatively were also identified.

**Conclusions:**

These data, for the first time, presented the temporal gene expression profiling and reveal the important process of molecular biology in the early-stage of traumatic TMJ bony ankylosis. The findings might contributed to identifying potential targets for the treatment of TMJ ankylosis.

**Supplementary Information:**

The online version contains supplementary material available at 10.1186/s12903-024-03971-x.

## Background

Temporomandibular joint (TMJ) ankylosis, characterised by a progressive limitation of mouth opening, is a pathological condition in which the mandible condyle is fused to the glenoid fossa by fibrous or bony tissues [1]. Traumatic TMJ ankylosis—the predominant form of the disease—is one of the most serious sequelae secondary to TMJ trauma, which not only affects the morphology of the oral and maxillofacial region, but also leads to serious dysfunction and decreased quality of life [2–4]. Considering the technical difficulties of surgery and the high incidence of recurrence, an in-depth understanding of the molecular pathophysiology of the disease is imperative.

The development of traumatic TMJ ankylosis is essentially a variation of bone healing, especially similar to delayed bone healing or hypertrophic nonunion [5]. The course from TMJ trauma to ankylosed joint is a highly sophisticated regenerative process, in which the early-stage inflammatory response and cell adhesion [6], the committed differentiation of mesenchymal stem cells [7], the changed mechanical stress [1, 8], the complex biological pathways and signaling molecules [1, 8–10], and the presumed genetic predisposition [1] could interact with each other.

Intra-articular haematoma organisation and ossification have been hypothesised as key processes in the pathogenesis of the disease [1]. New bone formation between the two traumatised articular surfaces is attributed primarily to endochondral ossification [11–13]. In histology, three phases have been confirmed in a sheep model: a fibrous-chondral phase in the first month, a chondral-calcified cartilage phase from the first to the third month, and a bone-cartilage phase from the third to the sixth month [9, 13]. Based on the findings from animal studies, ankylosis will inevitably occur once the traumatic microenvironment (i.e., damage to the physical barriers between the two articular surfaces) [14, 15] presents. Investigating the molecular biology underpinning the early stage of the disease (i.e., the fibrous-chondral phase during the first month) is especially crucial for discovering a new way to prevent the disease.

Previous studies have demonstrated that several important genes that regulate bone formation and angiogenesis (such as vascular endothelial growth factor (VEGF), VEGF receptor 2 (VEGFR2), Ang1, CYR61, Wnt and BMP signaling) are involved in the pathogenesis of the disease at several specific time points [8, 9, 16]. Recently, we put forward a comprehensive elucidation of the role of haematoma in the onset of traumatic TMJ ankylosis through a comparison of differential gene transcription profiles between the groups of haematoma absorbance and haematoma organisation at Days 1 and 4 postoperatively in a sheep model [17]. However, to the best of our knowledge, there are no published reports on genome-wide temporal transcriptional analysis of the entire fibrous–chondral phase of traumatic TMJ ankylosis. The aim of this study is to investigate the molecular biology underpinning the early stage of the disease by performing temporal gene expression profiling in a sheep model.

## Materials and methods

### Animal model and tissue processing

**Fig. 1:**
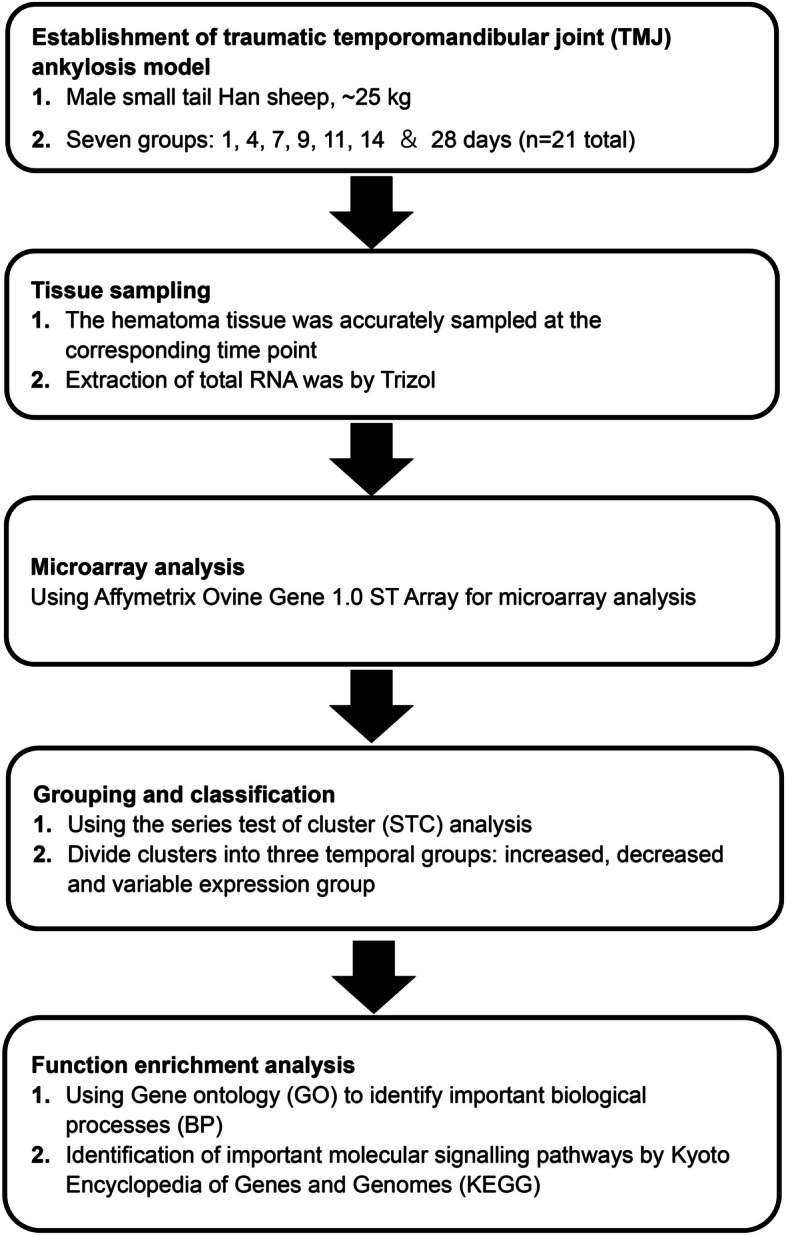
Flow chart of the chronological steps in the microarray analysis. Each box and the corresponding arrows show the main steps of the experimental design and genome-wide analysis using microarrays and gene expression analysis resources

The experiment was approved by the Ethics Committee of Tianjin Stomatological Hospital (approval number: Tjskq2013001). Twenty-one three-month-old male small-tailed Han sheep with an average weight of 23.5 ± 1.9 kg were used in this study. The housing and husbandry conditions of the animals, including breeding, light–dark cycle, room temperature, water quality and food, were as described in a previous study [14]. The animals received unilateral TMJ surgery—i.e., sagittal fracture of the condyle, removal of 2/3 of the articular discs and severely damaged articular fossa—to induce bony ankylosis, as performed in previous studies [13, 16]. The animals were sacrificed via euthanasia on Days 1, 4, 7, 9, 11, 14 and 28 after surgery, with three animals killed per time point (Fig. 1). The animals were euthanized in the same manner as in our previous study [6], that is, euthanized with a lethal dose of pentobarbital sodium (120 mg/kg) through the external jugular vein, and animal death was confirmed by observing respiratory and heartbeat arrest and loss of pupillary light and nerve reflexes. The haematoma or newly generated ankylosed callus in the joint spaces was harvested as described in a previous study [17].

A section of the harvested tissue from each sacrificed animal was immediately frozen in liquid nitrogen and then transferred to − 80 ℃ liquid nitrogen for ribonucleic acid (RNA) extraction and subsequent microarray analysis. The remaining tissue was fixed in 10% natural buffered formalin for 72 h, dehydrated, embedded in paraffin, and then cut with a microtome into 5 μm thick sections. Haematoxylin and eosin (HE) staining was performed, and the slides were observed under an Olympus BX51 microscope.

### RNA preparation, microarray hybridisation and data preprocessing

The RNA preparation and microarray assay were performed by the CNKINGBIO Corporation (Beijing, China), as described in previous studies [6, 17]: TRIzol reagent (Invitrogen Life Technologies, Carlsbad, USA) was used to extract total RNA, and a RNEasy small kit (Qiagen, Valencia, USA) was used to purify the extracted total RNA. Gene expression was analysed using the Affymetrix Ovine Gene 1.0 ST Array (Affymetrix, Santa Clara, CA, United States), which comprised over 22,047 known transcripts and expressed sequence tags. The arrays were scanned using a GeneChip® Scanner 3000 7G enabled for high-resolution scanning. Data acquisition from the microarrays also required the Affymetrix® GeneChip Command Console (AGCC) software. The raw expression data were first background corrected and quantile normalised by a robust multichip analysis (RMA) algorithm using default Affymetrix analysis settings. The values presented were in log2 RMA signal intensity.

### Identification of differentially expressed genes (DEGs)

The random variance model (RVM) t-test was used to identify differentially expressed genes (DEGs) in the microarray data analysis. The RVM t-test was used to filter DEGs by virtue of the advantage in small samples, which can improve the degree of freedom effectively [18, 19]. Taking the first day after surgery as the baseline, a significance analysis of microarrays (SAM) software was used to identify significantly differentiated expression, with a cut-off fold change value of > 2 and *P* < 0.05 at each time point. The *P* values were adjusted using the false discovery rate (FDR).

### Series test of cluster (STC) analysis

The STC analysis was used to describe the gene expression time series and the set of clusters most likely to have generated the observed time series [20, 21]. Because the signal density change tendencies of genes are different under different situations, we clustered short time series gene expression data into clear and definite unique profiles; genes clustered together in this way are likely to share similar physiological functions or regulation. The Cluster and TreeView software programs from Stanford University (Palo Alto, CA, USA) were used to perform the STC analysis based on the DEGs. The log2 fold change ratios were clustered using hierarchical clustering with a centred correlation distance or similarity metric and the average linkage clustering method. Based on the various expression trends, a cluster was assigned to one of three temporal expression groups. If the expression profiles of all the genes in a cluster showed a pattern of increased or decreased expression all the time, the cluster was assigned to the *increased expression group* or the *decreased expression group*. However, if the expression profiles of all the genes in a cluster exhibited patterns of both increased and decreased expression temporally, and the log2 fold changes in the expression values were less than 0.5 for all time points, the cluster was assigned to the *variable expression group*.

### Function enrichment analysis

Genes in three temporal time groups were further analysed using the Database for Annotation, Visualization and Integration Discovery (DAVID) version 6.8 to identify significant gene ontology categories and biological signaling pathways [22]. Gene Ontology (GO) term enrichment [23] was used to group the genes in the three temporal time groups into defined categories of biological process (BP). The overall major BP categories were formed by manually combining specific subcategory terms with related or overlapping functions. DAVID was also used to analyse the genes in each temporal expression group and the DEGs at each time point to find significant signaling pathways using Kyoto Encyclopedia of Genes and Genomes (KEGG) pathway maps [24]. The GO and KEGG pathway enrichment analyses were selected using Fisher’s exact test and χ2 tests. The standard of difference screening was *P* < 0.05.

## Results

### Representative histological images at each time point

**Fig. 2:**
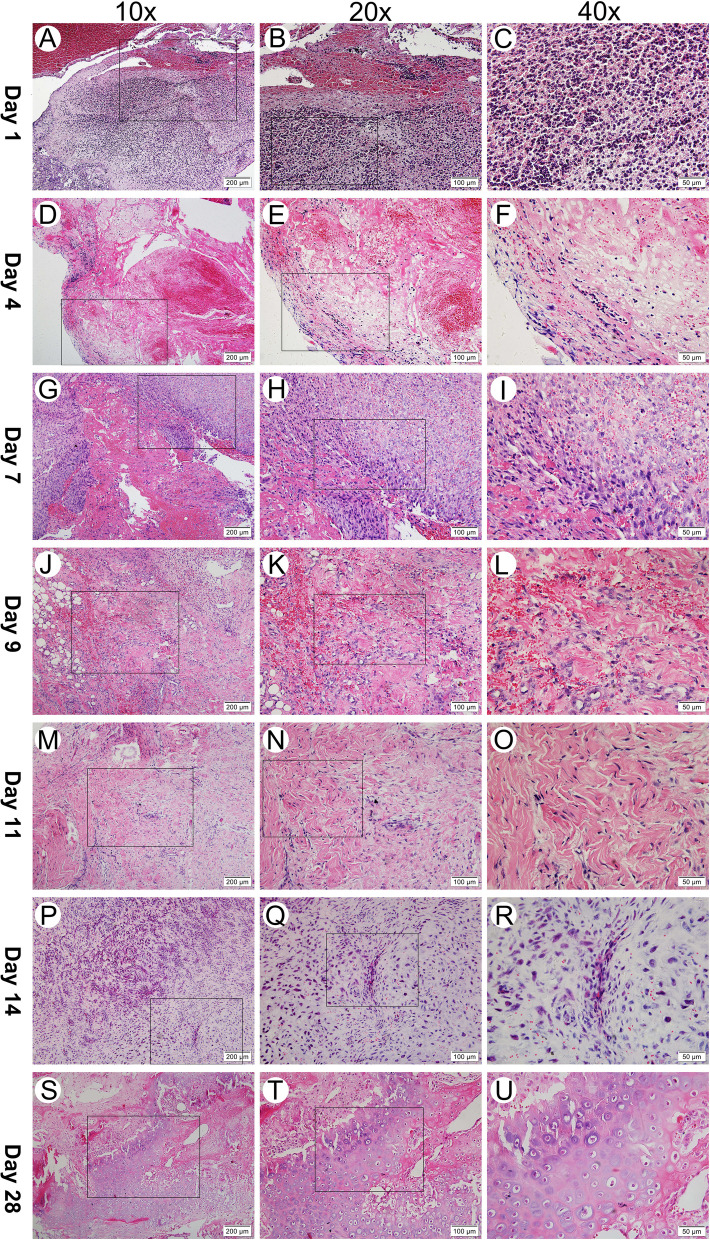
Histological observation of temporomandibular joint intra-articular hematoma or newly generated ankylosed callus at each post-operation time point (haematoxylin and eosin stain: 10 × 200 μm, 20 × 100 μm and 40 × 50 μm). Day 1: Blood clots containing a large number of neutrophils filled the articular space. Day 4: The apparent inflammatory cells subsided, and fibroblasts appeared for the first time. Day 7: The inflammatory cells disappeared completely, and organisation of the blood clots commenced, with cell transformations in a fibrovascular structure with small vessels and an immature collagenous network. Day 9 & Day 11: fibroblasts and a large number of red-stained collagen bundles were observed. Day 14 & Day 28: Cartilage formed locally and was characterised by fibrocartilage

The histological results for traumatic TMJ ankylosis in the first month in the sheep model are presented in Fig. 2. This fibrous-chondral formation sequence occurs in spatially and temporally complex domains within regions between the two traumatised articular surfaces. The process can be divided into four subphases: *inflammation subsidence phase* (Days 1–4), *granulation formation phase* (Days 4–7), *fibroblast proliferation phase* (Days 7–14), and *cartilage formation phase* (Days 14–28).

The Day-1 tissue samples had a fibrin network, platelets and many inflammatory cells (Fig. 2A–C). On Day 4, erythrocytes and the fibrin network were still obvious, fibroblasts began to appear at the edge of the haematoma, and there was an apparent decrease in the number of inflammatory cells (Fig. 2D–F). On Day 7, fibrovascular progenitors rapidly invaded and occupied most of the visual field; new capillaries could be detected, and there was still some residual fibrin scaffold, but there was no newly generated collagenous fibre (Fig. 2G–I). On Day 9, a mass of loosened collagenous fibre could be observed, interacting with a considerable number of new blood vessels (Fig. 2J–L). On Day 11, the collagenous fibre matured, becoming dense and coarse (Fig. 2M–O). On Day 14, a small quantity of round chondroid cells appeared in an avascular area near the area of plentiful angiogenesis (Fig. 2P–R). On Day 28, cartilage had formed locally, abundant hypertrophic chondrocytes and a cartilage matrix could be seen—which featured fibrocartilage, as the chondrocytes were separated by conspicuous fibrous bands (Fig. 2S–U).

### Overview of gene expression at each time point of TMJ ankylosis formation

**Fig. 3:**
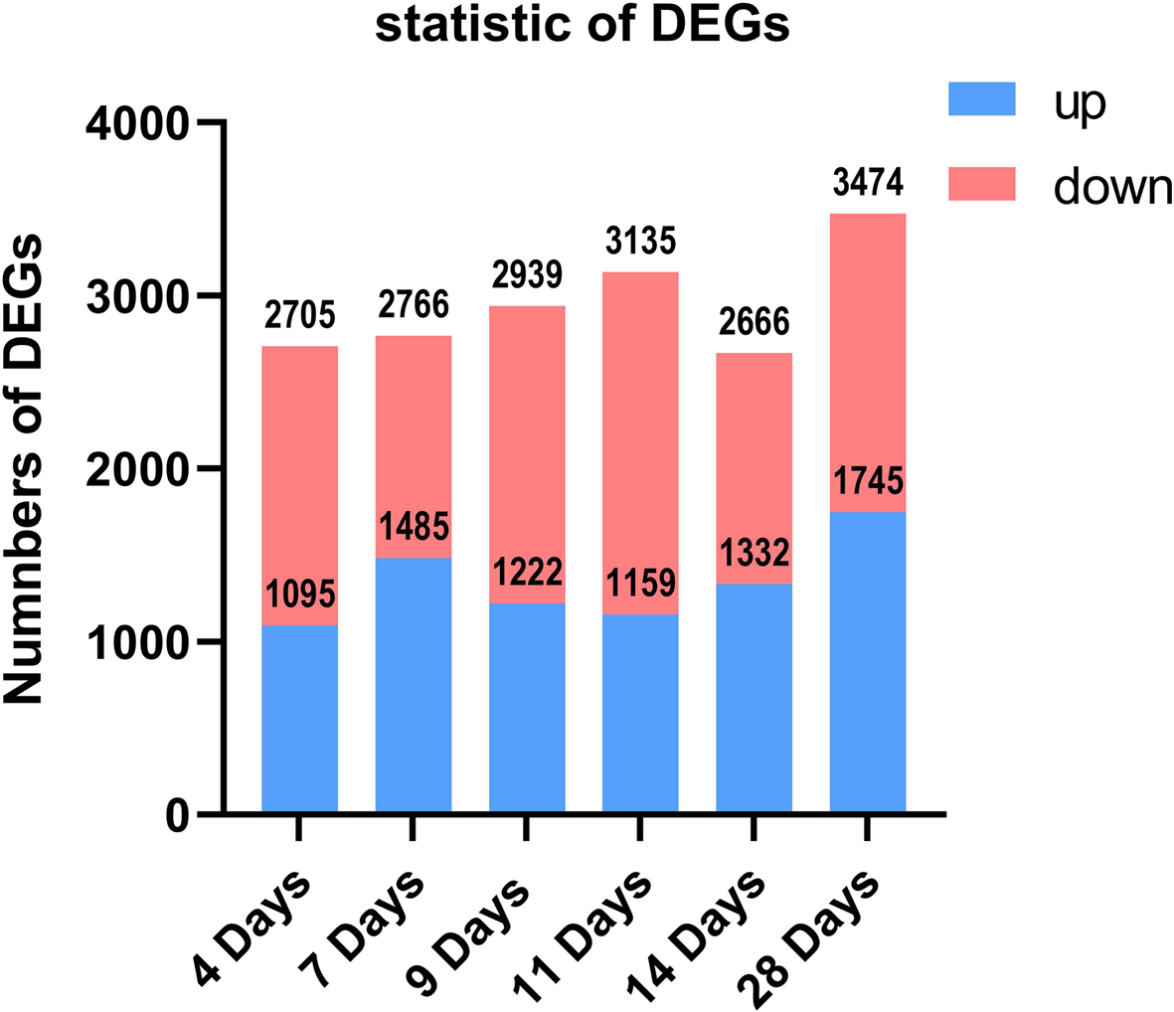
Stacked bar chart of the number of upregulated (blue) and downregulated (red) DEGs at each post-operation time point, with Day 1 as the control. DEGs, differentially expressed genes

A total of 5,087 mRNAs out of 22,142 genes showed altered levels of expression across the time course of a non-log transformed value of greater than ± 2 compared to the corresponding value on the first day after surgery. Based on the observed values, approximately 30% of the genes were differentially regulated in the process of TMJ ankylosis. The stacked bar chart in Fig. 3 highlights the number of upregulated (blue) and downregulated (red) DEGs at each time point.

### Temporal clusters of expression profiles

**Fig. 4:**
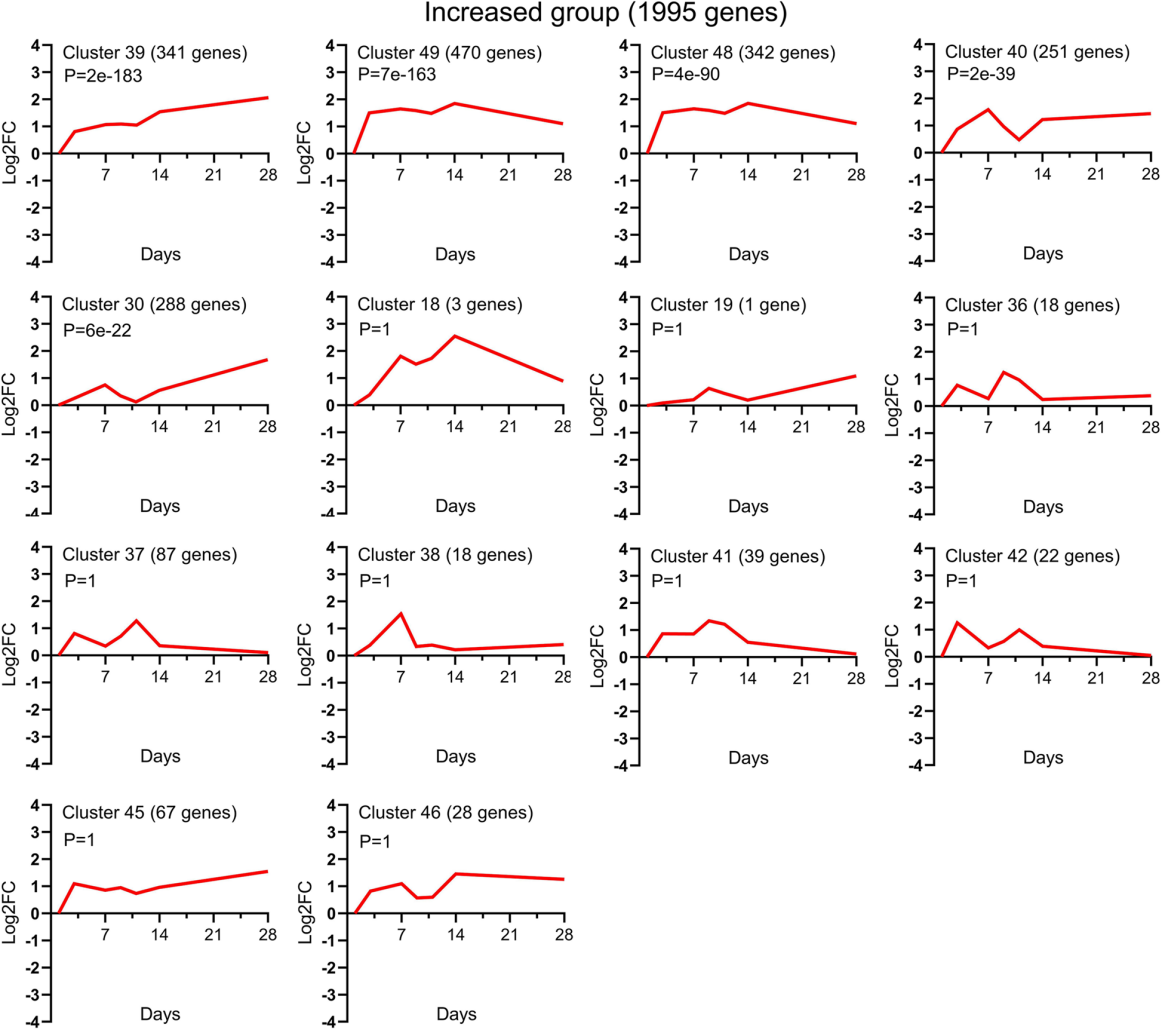
Temporal profiles of genes in the *increased expression group* across TMJ ankylosis. Using STC analysis, the expression profiles of 1,995 DEGs upregulated at all time points were clustered, and 14 temporal clusters were determined; among them, Clusters 39, 49, 48, 40 and 30 were statistically significant. The data is presented as log_2_ fold change values over the following time points: Day 1, 4, 7, 9, 11, 14 and 28 post operation. The cluster number, the number of genes for each clustered gene expression profile graph and the *P* value are specified in the title. STC, series test of cluster; DEGs, differentially expressed genes

**Fig. 5:**
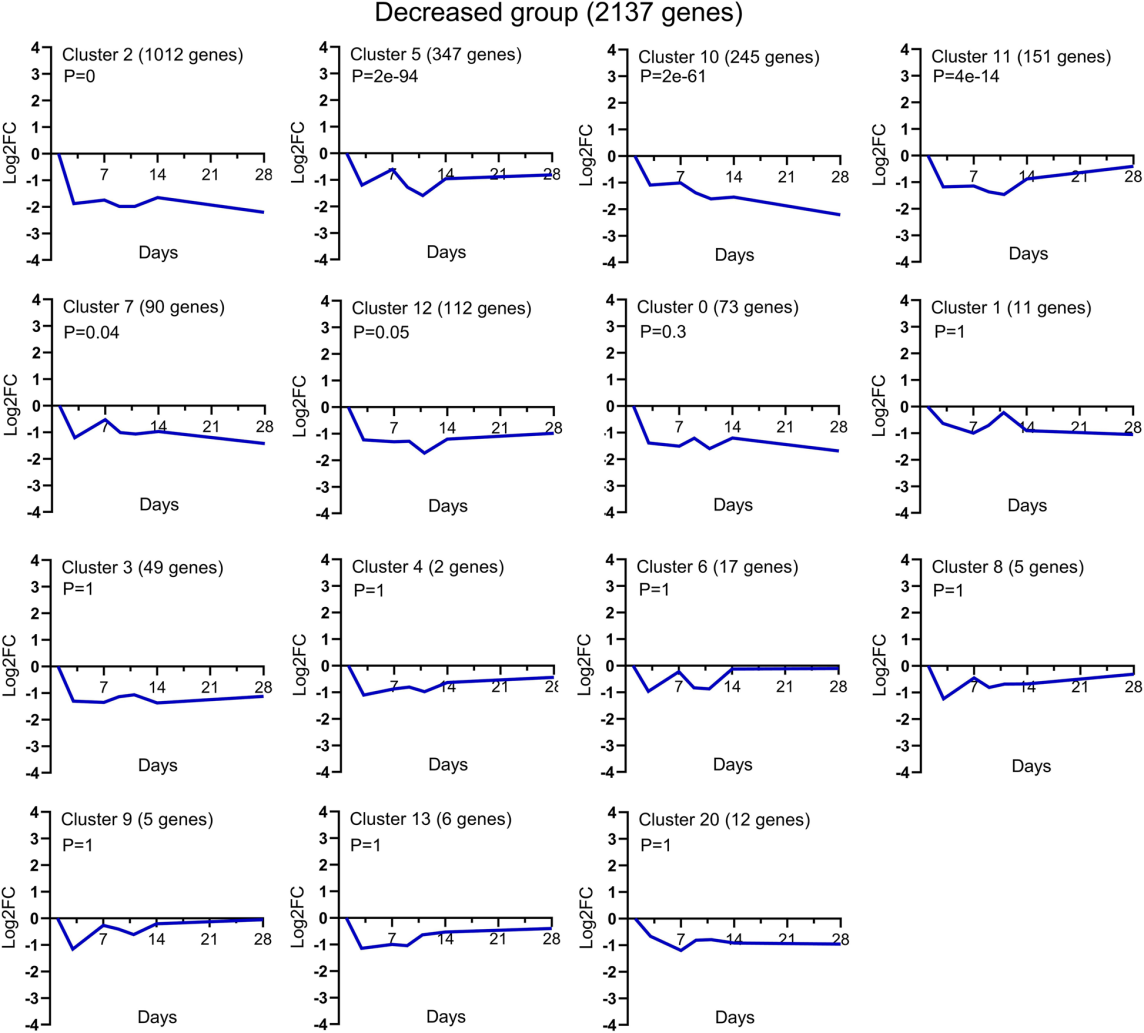
Temporal profiles of genes in the *decreased expression group* across TMJ ankylosis. Using STC analysis, the expression profiles of 2,137 DEGs downregulated at all time points were clustered, and 15 temporal clusters were obtained; among them, Clusters 2, 5, 10, 11 and 7 were statistically significant. The data is presented as log_2_ fold change values over the following time points: Day 1, 4, 7, 9, 11, 14 and 28 post operation. The cluster number, the number of genes for each clustered gene expression profile graph and the *P* value are specified in the title. STC, series test of cluster; DEGs, differentially expressed genes

**Fig. 6:**
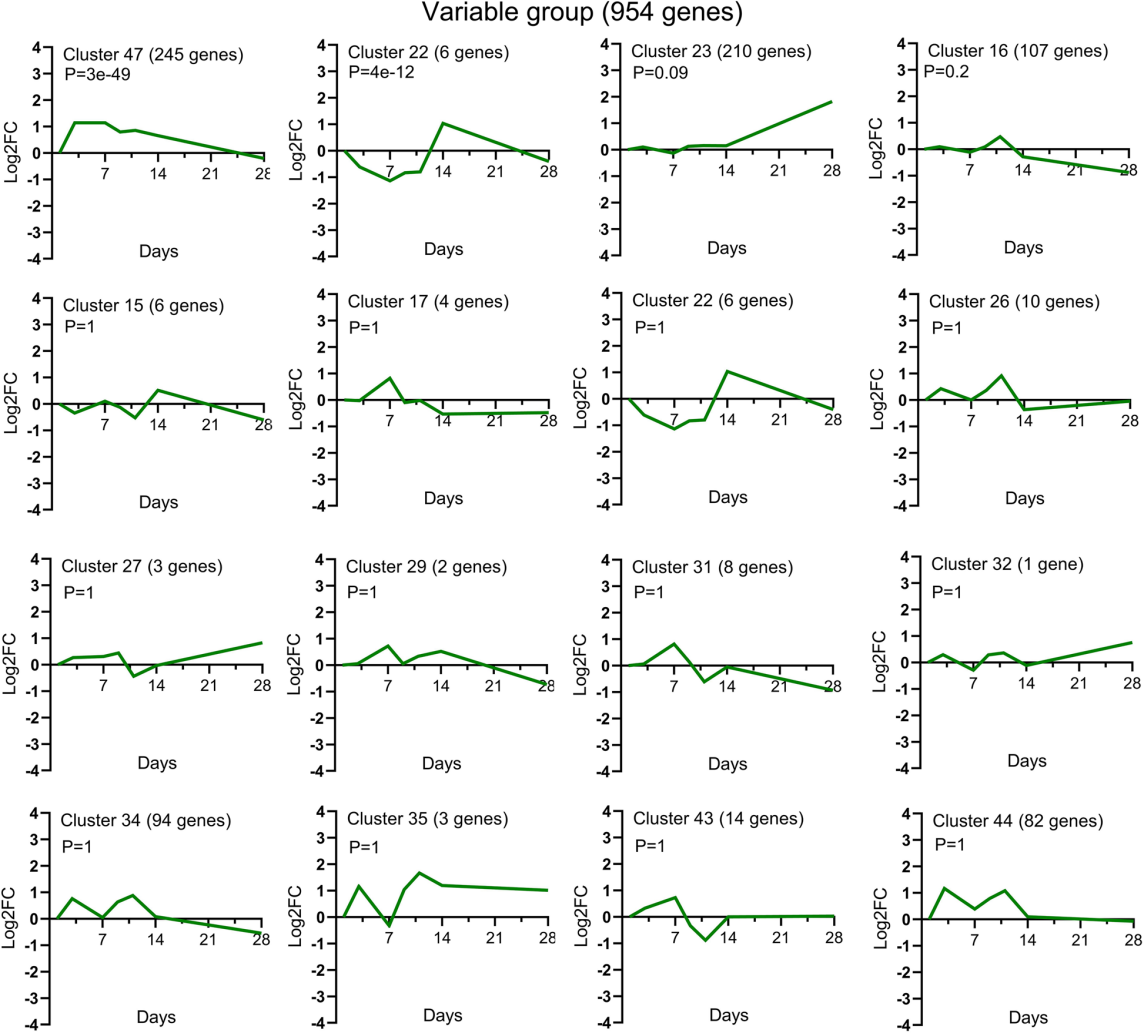
Temporal profiles of genes in the *variable expression group* across TMJ ankylosis. Using STC analysis, the expression profiles of 954 DEGs that temporally exhibited patterns of both increased and decreased expression were clustered, and 16 temporal clusters were obtained; among them, Clusters 47 and 22 were statistically significant. The data is presented as log_2_ fold change values over the following time points: Day 1, 4, 7, 9, 11, 14 and 28 post operation. The cluster number, the number of genes for each clustered gene expression profile graph and the P value are specified in the title. STC, series test of cluster; DEGs, differentially expressed genes

The graphical representations of the average expression profile of each of the 45 unique temporal clusters determined via STC analysis of significant gene probe sets are presented in Figs. 4, 5 and 6. Fourteen clusters were assigned to the increased expression group (Fig. 4); of these, Clusters 39, 49, 48, 40 and 30 were statistically significant. Fifteen clusters were assigned to the decreased expression group (Fig. 5); of these, Clusters 2, 5, 10, 11 and 7 were statistically significant. Of the 16 clusters assigned to the variable expression group (Fig. 6), Clusters 47 and 22 were statistically significant. The specific gene names for each temporal group and their corresponding clusters can be found in Supplementary file 1.

### Identification of significant BP and pathways associated with major temporal groups of clustered gene expression profiles

**Fig. 7:**
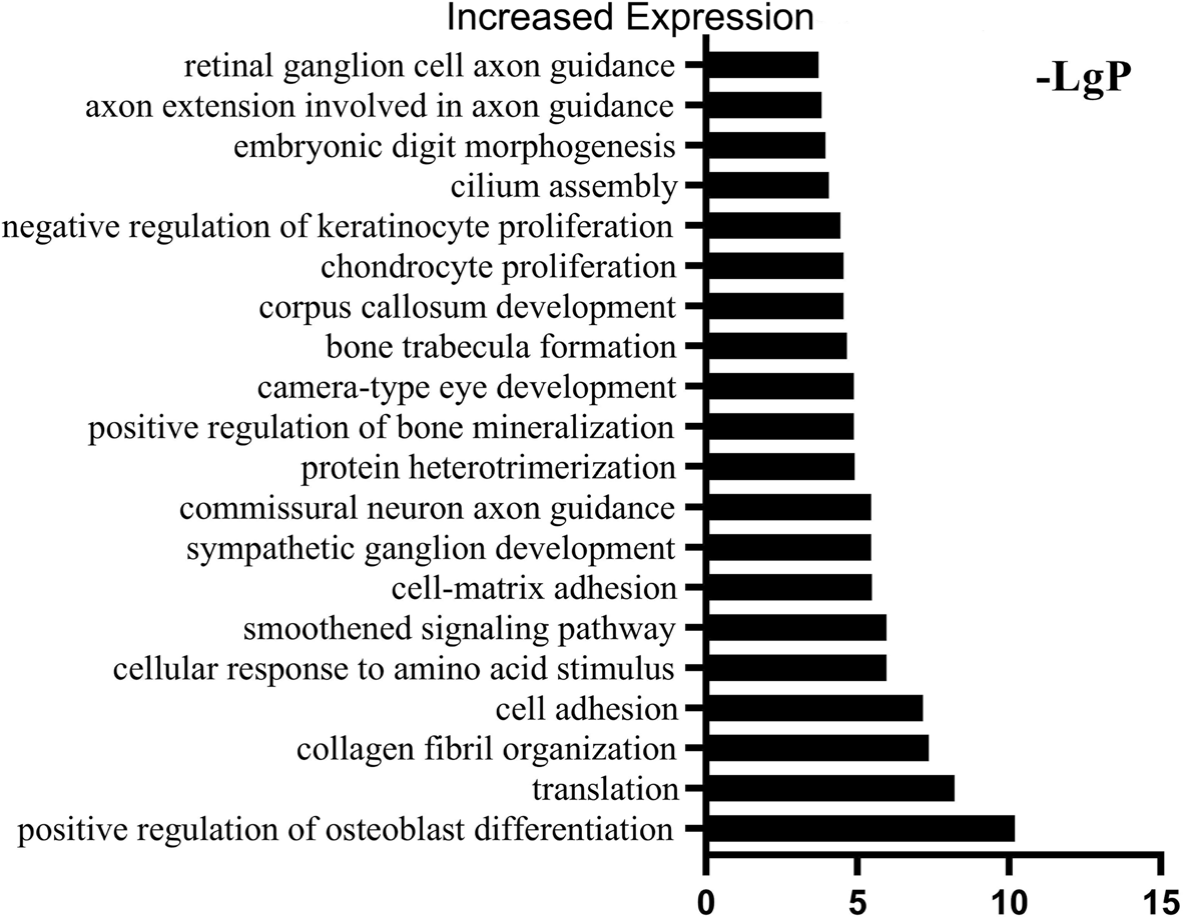
GO analysis of DEGs in the *increased expression group* identified the top 20 statistically significant BP terms in the *increased expression group*. The statistical significance is shown on the X-axis in − LgP. The Y-axis represents the names of the enriched pathway. GO, gene ontology; DEGs, differentially expressed genes

**Fig. 8:**
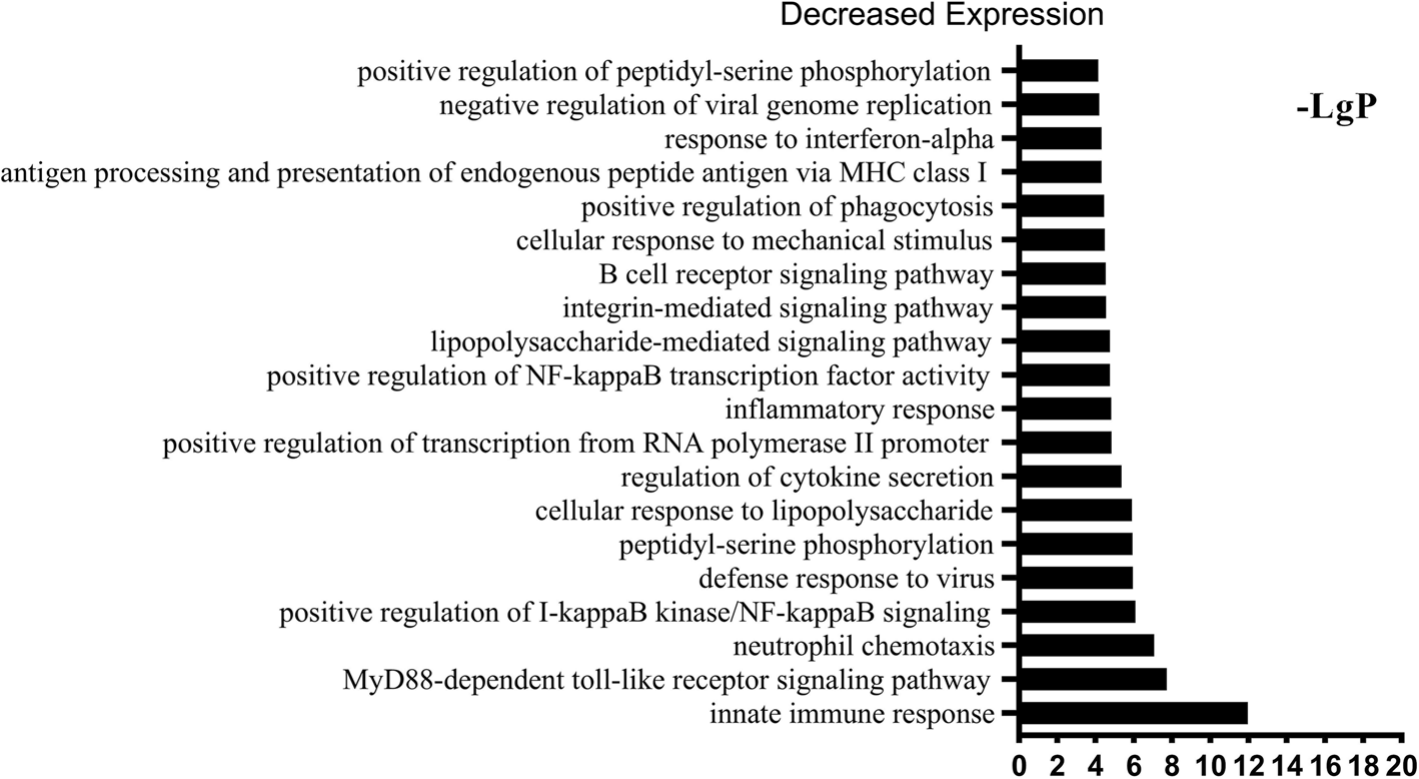
GO analysis of DEGs in the *decreased expression group* identified the top 20 statistically significant BP terms in the *decreased expression group*. The statistical significance is shown on the X-axis in − LgP. The Y-axis represents the names of the enriched pathway. GO, gene ontology; DEGs, differentially expressed genes

**Fig. 9:**
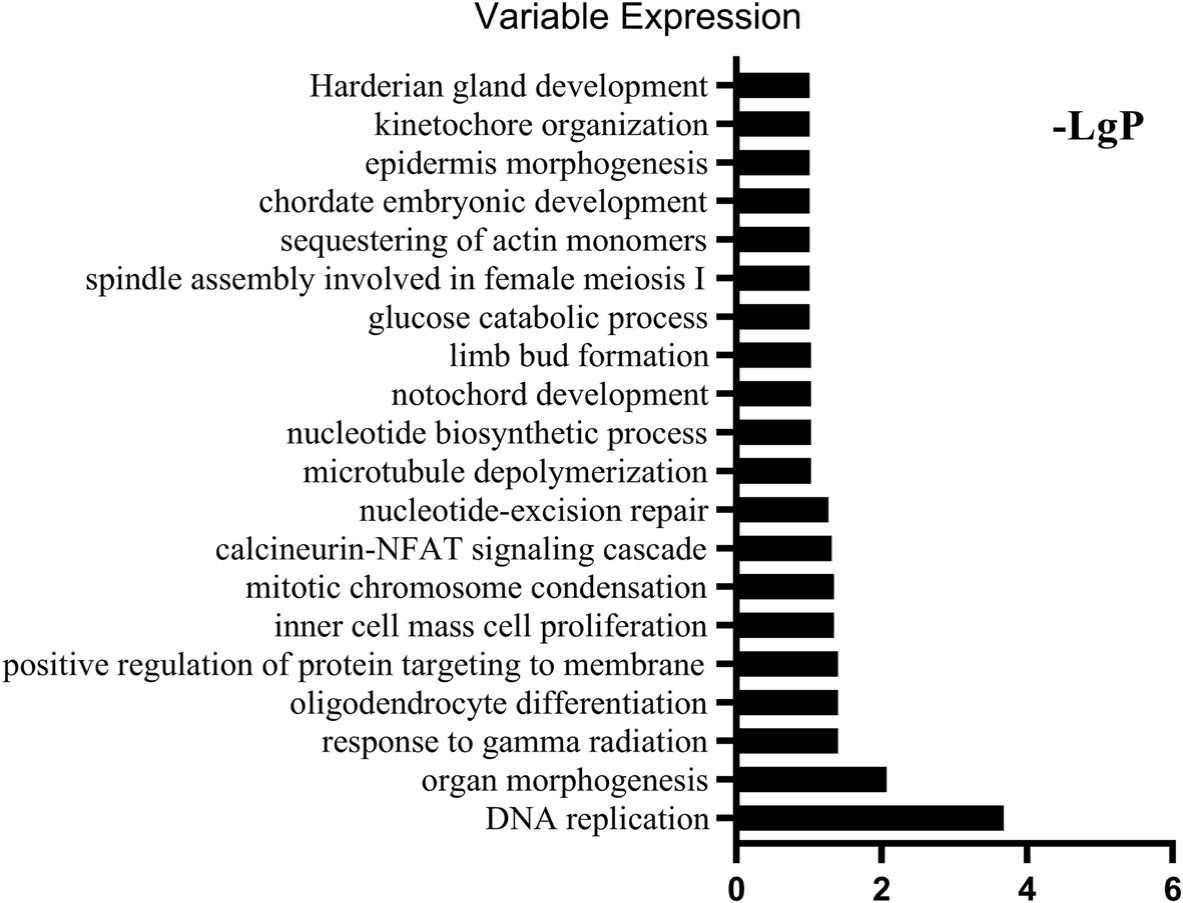
GO analysis of DEGs in the *variable expression group* identified the top 20 statistically significant BP terms in the *variable expression group*. The statistical significance shown on the X-axis is represented by − LgP. The Y-axis represents the names of the enriched pathway. GO, gene ontology; DEGs, differentially expressed genes

The column charts in Figs. 7, 8 and 9 presented respectively the top 20 statistically significant (*p* < 0.05) BP terms identified for the increased, decreased and variable expression groups. The details of each temporal group were summarised in Supplementary file 2. The BP identified for the *increased expression group* (Fig. 7) were predominantly elements of osteogenesis, including positive regulation of osteoblast differentiation (GO:0045669), collagen fibril organisation (GO:0030199) and chondrocyte proliferation (GO:0035988). In addition, the majority of the BP terms identified for the *decreased expression group* (Fig. 8) were related to immune and inflammatory responses, e.g., innate immune response (GO:0045087), MyD88-dependent toll-like receptor signaling pathway (GO:0002755), neutrophil chemotaxis (GO:0030593), positive regulation of I-kappaB kinase/NF-kappaB signaling (GO:0043123) and the B cell receptor signaling pathway (GO:0050853). The BP identified for the *variable expression group* (Fig. 9) comprised a large variety of metabolism-related processes, including DNA replication (GO:0006260), inner cell mass cell proliferation (GO:0001833) and cell aging (GO:0007569).

A KEGG pathway analysis of the genes in the three temporal expression groups identified the top 20 statistically significant signaling pathways (Table 1). This analysis showed that some of the critical pathways identified as unique to gene expression were present in only one of the three main temporal groups. Specifically, there are 47 significant signaling pathways in the *increased expression group*, including the Hippo signaling pathway, RAP 1 signaling pathway, Focal adhesion and Wnt signaling pathway (Table 1 and Supplementary file 3). This showed that the expressions of angiogenesis and osteogenesis were active at all temporal points. There were 114 critical signaling pathways in the *decreased expression group* (Table 1 and Supplementary file 3). There was a significantly high degree of osteoclast differentiation, and the activity of the NF-kappa B signaling pathway was elevated. Furthermore, many immune-related pathways were in the *decreased expression group*, including the nucleotide oligomerisation domain (NOD)-like receptor signaling pathway, Toll-like receptor signaling pathway, B cell receptor signaling pathway, T cell receptor signaling pathway and tumour necrosis factor (TNF) signaling pathway. This indicated that the inflammatory response and osteoclast differentiation peaked on Day 1 and then gradually subsided, which was consistent with our histological findings. In the *variable expression group*, there were 26 significant pathways, including DNA replication pathways, DNA repair and recombination pathways, cell cycle pathways, cell senescence pathways and related signal transduction and regulation pathways (Table 1 and Supplementary file 3).

**Table 1:** Pathway enrichment analysis in increased, decreased and variable expression groups

**Increased Expression Groups**	**Gene Amount in Pathway**	**Differentially Expressed Genes Counts in Pathway**	***P* value**
Rap1 signaling pathway	207	50	1.6333E-08
Axon guidance	178	45	1.9548E-08
Proteoglycans BR	55	21	7.3175E-08
Focal adhesion	209	46	1.0866E-06
ECM-receptor interaction	92	26	2.066E-06
Dilated cardiomyopathy (DCM)	85	24	5.1477E-06
Adherens junction	70	21	7.0027E-06
Arrhythmogenic right ventricular cardiomyopathy (ARVC)	68	20	1.599E-05
PI3K-Akt signaling pathway	372	66	1.8009E-05
Pathways in cancer	533	86	4.3892E-05
Hippo signaling pathway	155	33	7.019E-05
Hypertrophic cardiomyopathy (HCM)	82	21	9.5678E-05
Ras signaling pathway	230	43	1.55E-04
Phospholipase D signaling pathway	147	30	3.21E-04
Glycosaminoglycan binding proteins BR	214	39	5.22E-04
Parathyroid hormone synthesis, secretion and action	106	23	6.34E-04
Fluid shear stress and atherosclerosis	146	29	6.39E-04
Vascular smooth muscle contraction	140	28	7.02E-04
Ribosome	248	43	8.18E-04
cGMP-PKG signaling pathway	170	32	9.11E-04
**Decreased Expression Groups**	**Gene Amount in Pathway**	**Differentially Expressed Genes Counts in Pathway**	***P* value**
Measles	134	53	1.63E-15
Toll-like receptor signaling pathway	104	45	4.32E-15
Osteoclast differentiation	125	50	6.06E-15
Influenza A	183	63	8.96E-15
Chemokine signaling pathway	179	60	1.52E-13
NF-kappa B signaling pathway	93	40	1.84E-13
B cell receptor signaling pathway	67	33	2.17E-13
Epstein-Barr virus infection	226	69	3.98E-13
NOD-like receptor signaling pathway	175	58	6.68E-13
Hepatitis B	145	51	1.27E-12
C-type lectin receptor signaling pathway	103	40	9.61E-12
Acute myeloid leukemia	66	30	3.57E-11
Leishmaniasis	86	35	4.01E-11
Chronic myeloid leukemia	79	33	6.27E-11
Tuberculosis	200	59	9.60E-11
FoxO signaling pathway	133	45	1.19E-10
T cell receptor signaling pathway	100	37	2.97E-10
Kaposi sarcoma-associated herpesvirus infection	194	56	7.13E-10
Apoptosis	145	46	8.37E-10
Non-small cell lung cancer	64	27	2.64E-09
**Variable Expression Groups**	**Gene Amount in Pathway**	**Differentially Expressed Genes Counts in Pathway**	***P* value**
DNA replication proteins BR	130	22	2.58E-08
Chromosome and associated proteins BR	1207	86	8.85E-07
DNA repair and recombination proteins BR	316	32	4.66E-06
Cellular senescence	169	21	9.75E-06
Homologous recombination	45	10	1.47E-05
Cell cycle	130	17	3.52E-05
Prostate cancer	93	13	1.46E-04
Wnt signaling pathway	147	17	1.67E-04
Breast cancer	151	17	2.31E-04
Fanconi anemia pathway	53	9	3.53E-04
Basal cell carcinoma	65	10	3.85E-04
Gastric cancer	156	16	9.81E-04
DNA replication	39	7	1.13E-03
Glycosaminoglycan binding proteins BR	214	19	1.95E-03
Endometrial cancer	58	8	2.98E-03
Pancreatic cancer	76	9	4.78E-03
Hepatocellular carcinoma	174	15	7.32E-03
Bladder cancer	42	6	8.19E-03
Hepatitis B	145	13	8.83E-03
PI3K-Akt signaling pathway	372	26	8.90E-03

### Identification of significant pathways at each time point

Distinct from the critical pathways associated with the three temporal groups determined via clustering of differential genes, the significant pathways (*p* < 0.05) also need to be determined via analysis of the expression of important genes at each time point. Table 2 presented the biological pathways involved in differential gene expression, with subsequent levels of expression comparing against the expression level on the first day after surgery (Supplementary file 4). Over time, the cell activity in the up-regulated groups became increasingly complex with more critical pathways involving in, and the number of pathways were 33, 35, 42, 53, 54 and 61, respectively. However, this trend was not exhibited in the downregulated groups: the number of signal pathways involved at each time point was roughly the same, the number of pathways were 114,116,121,121,106, respectively (Supplementary file 4).

**Table 2:** The signaling pathways involved differentially expressed genes in Pathway at each time point compared with the first day after operation

Up	*P* value	Down	*P* value
**Day4 vs. Day**1			
Proteoglycans BR	8.73E-08	Measles	3.18E-17
Cell cycle	9.58E-07	Influenza A	2.13E-14
DNA replication proteins BR	3.65E-06	Chemokine signaling pathway	5.46E-13
ECM-receptor interaction	4.80E-06	C-type lectin receptor signaling pathway	7.05E-13
PI3K-Akt signaling pathway	1.16E-05	Leishmaniasis	1.90E-12
Rap1 signaling pathway	3.01E-05	Osteoclast differentiation	4.71E-12
Glycosaminoglycan binding proteins BR	1.40E-04	NOD-like receptor signaling pathway	1.26E-11
Focal adhesion	2.45E-04	Epstein-Barr virus infection	1.62E-11
Hypertrophic cardiomyopathy (HCM)	3.12E-04	NF-kappa B signaling pathway	2.16E-11
Dilated cardiomyopathy (DCM)	4.47E-04	Toll-like receptor signaling pathway	2.61E-11
Arrhythmogenic right ventricular cardiomyopathy (ARVC)	7.47E-04	B cell receptor signaling pathway	4.71E-10
Axon guidance	1.03E-03	FoxO signaling pathway	6.59E-10
Regulation of actin cytoskeleton	2.03E-03	Tuberculosis	1.70E-09
Hippo signaling pathway-multiple species	2.82E-03	Kaposi sarcoma-associated herpesvirus infection	1.86E-09
Hippo signaling pathway	3.09E-03	Pattern recognition receptors BR	2.47E-09
Oocyte meiosis	3.70E-03	CD molecules BR	4.93E-09
Cytoskeleton proteins BR	5.38E-03	Hepatitis B	8.85E-09
Chromosome and associated proteins BR	5.49E-03	Fc epsilon RI signaling pathway	1.08E-08
Protein kinases BR	7.56E-03	MAPK signaling pathway	9.79E-08
Gap junction	9.15E-03	Toxoplasmosis	1.00E-07
**Day7 vs. Day1**			
Proteoglycans BR	2.06E-08	Measles	2.78E-16
Axon guidance	1.40E-06	Influenza A	1.11E-15
Rap1 signaling pathway	6.69E-06	Chemokine signaling pathway	2.05E-15
Cell cycle	1.02E-05	C-type lectin receptor signaling pathway	5.43E-15
PI3K-Akt signaling pathway	4.74E-05	Toll-like receptor signaling pathway	4.91E-14
Focal adhesion	5.01E-05	Osteoclast differentiation	1.26E-13
ECM-receptor interaction	9.84E-05	NF-kappa B signaling pathway	3.58E-13
Hippo signaling pathway-multiple species	2.19E-04	B cell receptor signaling pathway	4.97E-13
DNA replication proteins BR	2.30E-04	Epstein-Barr virus infection	6.56E-12
Chromosome and associated proteins BR	8.06E-04	NOD-like receptor signaling pathway	6.69E-12
Regulation of actin cytoskeleton	9.21E-04	Leishmaniasis	9.54E-12
Pathways in cancer	9.93E-04	CD molecules BR	1.30E-11
Glycosaminoglycan binding proteins BR	1.73E-03	Hepatitis B	1.76E-11
Ras signaling pathway	2.74E-03	MAPK signaling pathway	2.71E-11
Dilated cardiomyopathy (DCM)	2.76E-03	Toxoplasmosis	1.04E-10
Hypertrophic cardiomyopathy (HCM)	5.12E-03	Pattern recognition receptors BR	2.77E-10
Protein kinases BR	5.27E-03	Kaposi sarcoma-associated herpesvirus infection	8.03E-10
N-Glycan biosynthesis	6.32E-03	Cytokine receptors BR	4.36E-09
Gap junction	6.61E-03	FoxO signaling pathway	8.27E-09
Arrhythmogenic right ventricular cardiomyopathy (ARVC)	7.05E-03	T cell receptor signaling pathway	1.18E-08
**Day9 vs. Day1**			
Proteoglycans BR	1.99E-08	Measles	9.22E-17
Rap1 signaling pathway	5.52E-07	Influenza A	1.26E-15
Focal adhesion	6.91E-07	Osteoclast differentiation	3.31E-15
ECM-receptor interaction	3.10E-06	Chemokine signaling pathway	3.19E-14
Axon guidance	5.16E-06	NF-kappa B signaling pathway	1.52E-13
PI3K-Akt signaling pathway	1.39E-05	B cell receptor signaling pathway	1.70E-13
Dilated cardiomyopathy (DCM)	6.13E-05	C-type lectin receptor signaling pathway	1.93E-13
Hypertrophic cardiomyopathy (HCM)	1.45E-04	Toll-like receptor signaling pathway	2.76E-13
Ribosome	1.65E-04	Leishmaniasis	3.51E-13
Arrhythmogenic right ventricular cardiomyopathy (ARVC)	2.51E-04	NOD-like receptor signaling pathway	7.41E-13
Glycosaminoglycan binding proteins BR	3.68E-04	Epstein-Barr virus infection	2.31E-11
Gap junction	5.99E-04	Hepatitis B	2.52E-11
Ribosome BR	7.88E-04	Tuberculosis	1.72E-10
Cytoskeleton proteins BR	8.77E-04	CD molecules BR	4.05E-10
Hippo signaling pathway-multiple species	1.33E-03	Toxoplasmosis	8.41E-10
Regulation of actin cytoskeleton	1.94E-03	T cell receptor signaling pathway	1.07E-09
Progesterone-mediated oocyte maturation	1.99E-03	FoxO signaling pathway	1.18E-09
Ras signaling pathway	2.37E-03	Acute myeloid leukemia	1.37E-09
Pathways in cancer	2.42E-03	Kaposi sarcoma-associated herpesvirus infection	1.77E-09
Vascular smooth muscle contraction	2.71E-03	TNF signaling pathway	2.60E-09
**Day11 vs. Day1**			
Rap1 signaling pathway	4.86E-09	Influenza A	6.08E-17
ECM-receptor interaction	2.86E-08	B cell receptor signaling pathway	3.96E-16
Proteoglycans BR	6.35E-08	Measles	1.73E-15
Focal adhesion	8.74E-07	Osteoclast differentiation	7.86E-15
PI3K-Akt signaling pathway	1.08E-06	Chemokine signaling pathway	2.15E-14
Axon guidance	3.97E-06	Toll-like receptor signaling pathway	4.91E-14
Glycosaminoglycan binding proteins BR	4.35E-06	Leishmaniasis	8.77E-13
Pathways in cancer	1.06E-05	Apoptosis	1.23E-12
Gap junction	1.73E-05	NOD-like receptor signaling pathway	5.36E-12
Platelet activation	2.25E-04	T cell receptor signaling pathway	7.33E-12
Progesterone-mediated oocyte maturation	2.83E-04	Epstein-Barr virus infection	1.33E-11
Dilated cardiomyopathy (DCM)	3.55E-04	NF-kappa B signaling pathway	1.39E-11
Relaxin signaling pathway	3.94E-04	Hepatitis B	1.89E-11
Wnt signaling pathway	5.01E-04	C-type lectin receptor signaling pathway	2.09E-11
Human papillomavirus infection	6.00E-04	Fc epsilon RI signaling pathway	3.46E-11
Hypertrophic cardiomyopathy (HCM)	8.88E-04	Acute myeloid leukemia	3.46E-11
Cell cycle	9.55E-04	FoxO signaling pathway	1.50E-10
Fluid shear stress and atherosclerosis	1.26E-03	TNF signaling pathway	1.61E-10
Vascular smooth muscle contraction	2.10E-03	Tuberculosis	4.67E-10
Arrhythmogenic right ventricular cardiomyopathy (ARVC)	2.25E-03	Kaposi sarcoma-associated herpesvirus infection	1.25E-09
**Day14 vs. Day1**			
Focal adhesion	6.42E-09	Measles	1.91E-17
Axon guidance	2.79E-08	Chemokine signaling pathway	4.74E-17
Rap1 signaling pathway	4.94E-08	CD molecules BR	4.26E-16
ECM-receptor interaction	7.32E-08	C-type lectin receptor signaling pathway	1.76E-15
PI3K-Akt signaling pathway	1.44E-07	Influenza A	3.03E-15
Hypertrophic cardiomyopathy (HCM)	3.41E-06	Epstein-Barr virus infection	5.34E-15
Arrhythmogenic right ventricular cardiomyopathy (ARVC)	3.67E-06	NF-kappa B signaling pathway	1.61E-14
Pathways in cancer	5.74E-06	Leishmaniasis	6.16E-14
Dilated cardiomyopathy (DCM)	6.01E-06	Toll-like receptor signaling pathway	1.03E-13
Proteoglycans BR	2.11E-05	B cell receptor signaling pathway	1.09E-13
Vascular smooth muscle contraction	4.63E-05	NOD-like receptor signaling pathway	2.05E-13
Glycosaminoglycan binding proteins BR	4.73E-05	Osteoclast differentiation	1.43E-12
Regulation of actin cytoskeleton	1.23E-04	Kaposi sarcoma-associated herpesvirus infection	9.96E-12
cGMP-PKG signaling pathway	1.63E-04	T cell receptor signaling pathway	3.43E-11
Hippo signaling pathway	2.38E-04	Pattern recognition receptors BR	5.83E-11
Gap junction	2.62E-04	Tuberculosis	1.09E-10
Adherens junction	3.45E-04	Toxoplasmosis	2.02E-10
Relaxin signaling pathway	3.93E-04	FoxO signaling pathway	2.19E-10
Ras signaling pathway	4.18E-04	Fc epsilon RI signaling pathway	2.40E-10
Hippo signaling pathway-multiple species	5.45E-04	Hepatitis B	6.48E-10
**Day28 vs. Day1**			
Proteoglycans BR	2.22E-10	Measles	6.65E-16
PI3K-Akt signaling pathway	3.95E-10	CD molecules BR	6.89E-14
Axon guidance	5.30E-10	Chemokine signaling pathway	1.74E-13
Rap1 signaling pathway	3.20E-09	Toll-like receptor signaling pathway	1.84E-12
Focal adhesion	1.32E-08	Osteoclast differentiation	2.52E-12
ECM-receptor interaction	2.26E-07	Epstein-Barr virus infection	2.96E-12
Adherens junction	1.11E-06	NF-kappa B signaling pathway	6.02E-12
Glycosaminoglycan binding proteins BR	1.47E-06	Influenza A	6.45E-12
Dilated cardiomyopathy (DCM)	9.22E-06	Leishmaniasis	1.46E-11
Pathways in cancer	1.07E-05	Tuberculosis	6.92E-11
Ras signaling pathway	2.33E-05	C-type lectin receptor signaling pathway	1.54E-10
Wnt signaling pathway	4.07E-05	NOD-like receptor signaling pathway	1.75E-10
cGMP-PKG signaling pathway	4.95E-05	Pattern recognition receptors BR	2.59E-10
Phospholipase D signaling pathway	1.03E-04	B cell receptor signaling pathway	4.06E-10
Glycosaminoglycan biosynthesis-keratan sulfate	1.27E-04	Fc epsilon RI signaling pathway	8.81E-09
Relaxin signaling pathway	2.37E-04	Cytokine receptors BR	1.40E-08
Proteoglycans in cancer	5.03E-04	T cell receptor signaling pathway	2.28E-08
Regulation of actin cytoskeleton	5.09E-04	TNF signaling pathway	4.57E-08
Hypertrophic cardiomyopathy (HCM)	5.47E-04	Jak-STAT signaling pathway	4.57E-08
Hippo signaling pathway	6.13E-04	MAPK signaling pathway	9.17E-08

In the upregulated groups, 16 signaling pathways were coexpressed at each time point. Several pathways were interesting, e.g., the Wnt signaling pathway, Hippo signaling pathway and Rap1 signaling pathway, which were upregulated significantly (Table 2 and Supplementary file 4).

In the downregulated groups, most of the signaling pathways were coexpressed at all time points. Ninety-two signaling pathways were coexpressed at each time point; among them, the osteoclast differentiation signaling pathway was significantly downregulated at each time point. In addition, immune-related pathways, such as the Toll-like receptor signaling pathway, NOD-like receptor signaling pathway, B cell receptor signaling pathway, T cell receptor signaling pathway and TNF signaling pathway, were downregulated at all time points (Table 2 and Supplementary file 4).

## Discussion

In this study, we identified significant BP and pathways via genome-wide transcriptional analysis of the entire fibrous–chondral phase of traumatic TMJ ankylosis in a sheep model. Days 1, 4, 7, 9, 11, 14 and 28 were selected as time points based on the findings of previous studies [8, 9, 17] on the early stages of TMJ ankylosis include inflammation, hematoma organisation and TMJ fibrous-chondral ankylosis formation. The timeline was confirmed using serial histological sections and gene expression profiles. The DEGs were divided into 45 clusters via a cluster analysis and then subdivided into three temporal expression groups based on the expression trend (Figs. 4, 5 and 6 and Supplementary file 1). Subsequently, a list of the significant differentially expressed biological processes and signaling pathways among the three groups was used as a genome-wide overview of the main biological functions following ankylosis. The top 20 significant biological processes (Figs. 7, 8 and 9 and Supplementary file 2) and the top 20 critical signaling pathways (Table 1 and Supplementary file 3) were identified for the increased, decreased and variable expression groups to serve as a genome-wide overview of the main biological functions that occur during the entire fibrous-chondral phase of traumatic TMJ ankylosis. Identifying the significant pathways (Table 2 and Supplementary file 4) expressed at each time point post operation provides crucial insight into the regulation of TMJ ankylosis in a complete temporal context.

Based on the histological results, Days 1, 4, 7, 9, 11, 14 and 28 after surgery were divided into four substages: *inflammation subsidence phase* (Days 1–4), *granulation formation phase* (Days 4–7), *fibroblast proliferation phase* (Days 7–14) and the *cartilage formation phase* (Days 14–28). This regenerative sequence occurs in spatially and temporally complex domains within the intra-articular hematoma. However, due to the heterogeneity of time and space, each process overlaps the other, and during some periods, all the samples have similar performances. A significant number of gene chips combined with histological spatial graphics facilitate the identification of those genes that are particularly important for the formation of ankylosis but only appear in specific areas.

After surgery, intra-articular and peri-articular bleeding occurred immediately, and blood clots filled the intra-articular space. Dense inflammatory cell infiltration could be seen in the blood clots—primarily neutrophils. Subsequently, the number of inflammatory cells gradually decreased, indicating that the inflammation subsided gradually. This is the first stage, one to four days after surgery: the *inflammation subsidence phase* (Fig. 1A–F). This process has similar stages as fracture healing [25, 26].

Based on our previous study, we know that the development of TMJ ankylosis is similar to malunion of a fracture [5]. It has been suggested that transient and highly regulated secretion of proinflammatory molecules after acute injury is essential for tissue regeneration after fracture [27], with acute inflammatory responses peaking within the first 24 h and ending after seven days [28]. This is consistent with the results of our bioinformatics analysis: the majority of the BP identified via the *decreased expression group* (Fig. 8) are linked to immune and inflammatory responses. This indicates that in this study, there was a spike in inflammation on Day 1, followed by a gradual decline—similar to our previous study. Most of these immune and inflammatory responses are functions of innate immunity; among them, the MyD88-dependent toll-like receptor signaling pathway [29], its downstream signaling pathway [30], and the positive regulation of I-kappa B kinase/NF-kappa B signaling were dominant, which is consistent with the findings in our previous studies [6]. Because Toll-like receptors (TLRs) act as pattern-recognition receptors, they may play a critical role in recognising damage-related molecular patterns (DAMPs) and the TLR4/MyD88/NF-κB signaling pathway and its subsequent series of inflammatory reactions, which can promote the development of TMJ ankylosis. Furthermore, the NOD-like receptor signaling pathway was enriched, as indicated by the KEGG analysis of the *decreased expression group*. NOD-like receptors (NLRs) [31], which are also cytoplasmic pattern-recognition receptors (PRRs), can recognise DAMPs, triggering the activation of innate immunity. These results indicate that inhibition of the two signaling pathways (i.e., TLRs and NLRs) in the inflammatory phase of TMJ ankylosis might prevent the formation of ankylosis. The influx of inflammatory cells also leads to the secretion of chemokines such as IL6 (Cluster 10) and CXCR4 (Cluster 2). BMP4 and VEGF are released into the microenvironment, along with these proinflammatory chemokines, at the site of acute injury [32] and they interact with newborn bone progenitor cells to promote osteogenic differentiation.

In a temporal series analysis of rat fracture, most of the immune-related signaling pathways were classified into a *variable expression group* [33, 34], which differs from our experimental results. The reason is speculated to be the difference in the control groups used in each study. In the analysis of rat fracture, the researchers used normal bone marrow tissue as the control group [33, 34], while in this study, the control group was a hematoma on the first day after surgery.

Osteoclast differentiation signaling pathway was significantly enriched in the *decreased expression group*, which is a deviation from the fracture healing process and deserving of our attention. Osteoclasts, as macrophage lines, primarily receive inflammatory cytokines, such as Interleukin-1 (IL-1), Interleukin-6 (IL-6) and tumour necrosis factor α (TNF-α), from macrophages. These genes promote the formation and differentiation of osteoclasts and mediate the process of bone resorption. IL-1 activates TNF receptor associated factor 6 (TRAF6) molecules, which activate the downstream nuclear factor-kB (NF-κB). The importance of NF-κB in osteoclast formation has been reported in a previous study [35]. In our experiment, the expression of IL-1A (Cluster 0), IL-6 (Cluster 10), TNF-α (Cluster 2), TRAF6 (Cluster 5) and NF-κB (Cluster 4) were significantly downregulated at all time points, indicating that the formation and differentiation of osteoclasts were consistently inhibited (Tables 1 and 2). Although the inflammatory phase of fracture healing begins in the early stage, inflammatory factors are present throughout the entire repair process. The expression of IL-6 in the process of fracture healing exhibited a bimodal pattern, indicating that this inflammatory factor is time specific in the fracture healing process. In addition, other studies have shown that inflammation upregulates the expression of the proinflammatory cytokines TNF-α, IL-6 and IL-1 via the NF-kB pathway and is beneficial to normal fracture healing [36, 37]. In the final stage of fracture healing, bone tissue must be remodelled, and mature woven bone replaces lamellar bone, with osteoclasts playing a crucial role. Some researchers believe that ankylosis is the fusion of two similar damaged bone surfaces [38], which is analogous to faulty tissue differentiation after a fracture. This abnormal fusion and bone mass formation may stem from the inhibition of osteoclast formation and differentiation. Consequently, the final remodelling stage cannot be successfully initiated.

From the fourth to the seventh day after surgery, fibroblast-like cells that quickly invaded the blood clot began to release a collagen matrix to prepare for the formation of granulation tissue (Fig. 1D–I). This process is similar to the model of long bone fracture healing and plays the role of recruiting mesenchymal stem cells. At this point in the timeline, blood supply is insufficient due to the rupture of blood vessels in the traumatic microenvironment, resulting in hypoxia, acute cell necrosis and acidosis. Therefore, reconstruction of the blood supply is extremely important. At this point in the timeline, endothelial cells proliferate in the primitive collagen matrix, facilitating neovascularisation and interconnecting the capillary networks, which connect to peripheral blood vessels to re-establish circulation. With the establishment of circulation via a primitive vascular network, mast cells and monocytes multiplied, necrotic tissue gradually disappeared, vascular density and diameter increased, and fibroblasts proliferated and began secreting large amounts of collagen. At this point in the timeline, the granulation tissue became more mature (Fig. 1P–R). Histologically, these processes were classified as the *granulation formation phase* and the *fibroblast proliferation phase* in this study.

Focusing on the *granulation formation phase* and *fibroblast proliferation phase*, there were many genes involved in the angiogenesis expressed at this point. It is well known that the pathways regulating angiogenesis are the VEGF signaling pathway and the angiopoietin (Ang) signaling pathway [39]. The former is commonly referred to as VEGF A, and its receptor is VEGFR2 (also known as KDR), while the latter includes Ang1 (Angpt1), Ang2 (Angpt2) and the TEK tyrosine kinases receptor (TIE2). In this study, the differential genes did not include VEGFA, while VEGFR2, Angpt1, Angpt2, TIE2 (Cluster 48), VEGF B (Cluster 39) and VEGF C (Cluster 47) were persistently highly expressed at all time points.

The Hippo signaling pathway is highly conserved and plays a vital role in mediating organ development, tissue regeneration and self-renewal, and its expression is significantly upregulated at all time points (Tables 1 and 2). Yap1 is a component of the Hippo signaling pathway that has been shown in many studies to be closely linked to vascular regeneration. Yap1 (Cluster 49) was significantly highly expressed at each time point and peaked on Day 14 (Fig. 4). It has been reported that the retinal vascular density of mice decreases significantly after knockdown of Yap1; the development of cardiac valves was impaired and the mice died after Yap1 knockout [40]. Regarding the process of mouse retinal vascular development, some researchers have found that Yap1 regulates angiogenesis and remodelling via Ang2 activation [41]. In this study, Ang2, TIE2 and Yap1 are in the *increased expression group*, which indicates that ankylosis formation may be dominated by the Ang proteins and activated by the Hippo signaling pathway. In addition, Yap1 receptors include connective tissue growth factor (CTGF) and cysteine-rich angiogenic inducer 61 (CYR61), two genes that have been reported to be angiogenesis-related and involved in tumour angiogenesis [42]. In this study, CTGF and CYR61 (Cluster 45) were significantly upregulated at all time points (Fig. 4), indicating the importance of the Hippo signaling pathway in bone ankylosis formation.

In the *cartilage formation phase*, cartilage-like cells appeared for the first time, and the collagen matrix was also mineralised during this phase. Over time, there were increasingly more cartilage-like components (Fig. 1S–U).

Some genes that promote collagen matrix mineralisation, such as FMOD and LUM, reached peak expression during the *cartilage formation phase*, which is consistent with the findings of our previous study [17]. Furthermore, we found that the Wnt signaling pathway was significantly expressed at all time points in the TMJ ankylosis formation process (Table 1 and 2). This pathway has been shown to promote cell proliferation and mediate osteoblast differentiation in bone regeneration [43]. In our experiment, genes related to the Wnt pathway included WNT5A, Fzd1, Fzd3, Fzd6, Ctnnβ1 (β-catenin) and Ccnd2. This is similar to reports from two previous studies [33, 34]. One of these studies found that the temporal expression pattern of WNT5A increased on the first day, decreased on the third day, peaked on the fifth day, decreased again on the seventh to tenth day and then decreased to the baseline level on the fourteenth day. The other study found that the expression of WNT5A remained high and decreased at only five time points, peaking on the tenth day. The expression pattern of WNT5A in the model in our study was significantly different from that in the two studies mentioned earlier. The expression of the WNT5A gene (Cluster 48) increased from Day 1 to Day 7 after surgery, peaked at Day 7, decreased gradually from Day 7 to Day 11, peaked at Day 14 and exhibited a downward trend at Day 28 (Fig. 4). Wnt5a is an atypical Wnt ligand that signals independently of Ctnnβ1 and inhibits the typical Wnt pathway by degrading Ctnnβ1. Some studies have found that Wnt5a plays an important role in the early stages of fracture repair (inflammation and chondrogenesis) [44]. Mesenchymal stem cells (MSCs) migrate, proliferate and differentiate into chondroblasts or osteoblasts in the early stage of fracture repair, and studies have shown that the Wnt signaling pathway promotes the differentiation of MSCs into osteoblasts [45]. This indicates that the Wnt signaling pathway plays a significant role in ankylosis formation. Combined with histological observation, the early expression of Wnt5a may have two aspects: inhibiting inflammation and promoting the migration of MSCs to the injured area. Furthermore, the upregulation of expression on Day 14 combined with histological manifestations indicates that WNT5A may also promote the differentiation of MSCs into chondroblasts.

In this experiment, a large number of BP terms and signaling pathways related to cytoskeleton and cell adhesion were enriched in the increased expression group, such as the BP terms cell adhesion and cell–matrix adhesion, and the Rap1, Ras, Focal adhesion, ECM-receptor interaction and Adherens junction signaling pathways. Ras and Rap1 belong to the small molecular weight G protein of the Ras superfamily, which plays an important role in the regulation of cytoskeletal rearrangement, integrin function and other basic life activities, with Ras and Rap1 working together to initiate and maintain the ERK signal [46]. Some researchers have found that cytoskeletal modification, especially focal adhesion modification, is involved in the osteoblastic differentiation of MSCs [47], and is thought to be critical in the differentiation of osteoblasts on collagen-based substrates [48], with the cytoskeletal changes being mostly under the influence of mechanical forces. Therefore, in the future, the external force on the TMJ after trauma should be analyzed to further deepen our understanding of the pathogenesis of TMJ ankylosis.

## Conclusions

The data presented in this study capture the dynamic changes in intra-articular gene expression during an initial instance of TMJ ankylosis formation. The main observation is as follows: genes linked to osteoblast differentiation and angiogenesis are extensively involved in traumatic TMJ ankylosis formation, with several significant pathways, such as the Hippo pathway, Wnt signaling pathway and Rap 1 signaling pathway, being involved in this process. We also found that genes related to osteoclast differentiation and innate immunity during this process reached peak expression on the first day after surgery and were subsequently suppressed. The gene expression profiles enhance our understanding of the pathogenic mechanism underpinning TMJ ankylosis and present an opportunity to apply preventive molecular therapeutic targets prior to the development of irreversible TMJ bony ankylosis.

### Supplementary Information

**Additional file 1.**

**Additional file 2.**

**Additional file 3.**

**Additional file 4.**

## Data Availability

The datasets used and/or analysed during the current study are available from the corresponding author on reasonable request.

## Abbreviations

AGCC: Affymetrix® GeneChip Command Console
Ang/Angpt: Angiopoietin
β-catenin: Ctnnβ1
BP: Biological process
CTGF: Connective tissue growth factor
CYR61: Cysteine-rich angiogenic inducer 61
DAMPs: Damage-related molecular patterns
DAVID: Database for Annotation, Visualization and Integration Discovery
DEGs: Differentially expressed genes
FDR: False discovery rate
GO: Gene Ontology
HE: Haematoxylin and eosin
IL-1: Interleukin-1
IL-6: Interleukin-6
KEGG: Kyoto Encyclopedia of Genes and Genomes
MSCs: Mesenchymal stem cells
NF-κB: Nuclear factor-kB
NLRs: NOD-like receptors
NOD: Nucleotide oligomerisation domain
PRRs: Pattern-recognition receptors
RMA: Robust multichip analysis
RNA: Ribonucleic acid
RVM: Random variance model
SAM: Significance analysis of microarrays
STC: Series test of cluster
TIE2: TEK tyrosine kinases receptor
TLRs: Toll-like receptors
TMJ: Temporomandibular joint
TNF: Tumour necrosis factor
TNF-α: Tumour necrosis factor α
TRAF6: TNF receptor associated factor 6
VEGF: Vascular endothelial growth factor
VEGFR2: VEGF receptor 2
